# Reproduction of contagious bovine pleuropneumonia via aerosol-based challenge with *Mycoplasma mycoides* subsp. *mycoides*

**DOI:** 10.1186/s13028-020-00560-0

**Published:** 2020-11-16

**Authors:** Flavio Sacchini, Anne Mariana Liljander, Martin Heller, Elizabeth Jane Poole, Horst Posthaus, Elise Schieck, Joerg Jores

**Affiliations:** 1 International Livestock Research Institute, Old Naivasha Road, PO Box 30709, Nairobi, KE-00100 Kenya; 2 OIE Reference Laboratory for Contagious Bovine Pleuropneumonia, Istituto Zooprofilattico Sperimentale dell’Abruzzo e del Molise “G. Caporale”, Via Campo Boario, 64100 Teramo, Italy; 3 Friedrich-Loeffler-Institute—Federal Research Institute for Animal Health, Naumburger Str. 96a, 07743 Jena, Germany; 4 Institute of Animal Pathology, Department of Infectious Diseases and Pathobiology, Vetsuisse Faculty, University of Bern, Längassstrasse 122, CH-3012 Bern, Switzerland; 5 COMPATH, Vetsuisse Faculty & Faculty of Medicine, University of Bern, Längassstrasse 122, CH-3012 Bern, Switzerland; 6 Institute of Veterinary Bacteriology, Department of Infectious Diseases and Pathobiology, Vetsuisse Faculty, University of Bern, Längassstrasse 122, CH-3012 Bern, Switzerland

**Keywords:** Aerosol, CBPP, Contagious bovine pleuropneumonia, Infection model, Intranasal, *Mycoplasma mycoides* subsp. *mycoides*, Spray

## Abstract

Contagious bovine pleuropneumonia (CBPP) is a respiratory disease caused by *Mycoplasma mycoides* subsp. *mycoides*. Infection occurs via *Mycoplasma*-containing droplets and therefore requires close contact between animals. The current infection models are suboptimal and based on intratracheal installation of mycoplasmas or in-contact infection. This work tested the infection of adult cattle via aerosols containing live mycoplasmas mimicking the infection of cattle in the field. Therefore, we infected six cattle with aerosolized *Mycoplasma mycoides* subsp. *mycoides* strain Afadé over seven consecutive days with altogether 10^9^ colony forming units. All animals seroconverted between 11–24 days post infection and five out of six animals showed typical CBPP lesions. One animal did not show any lung lesions at necropsy, while another animal had to be euthanized at 25 days post infection because it reached endpoint criteria. Seroconversion confirmed successful infection and the spectrum of clinical and lesions observed mirrors epidemiological models and the field situation, in which only a fraction of animals suffers from acute clinical disease post infection.

## Findings

Contagious bovine pleuropneumonia (CBPP) caused by *Mycoplasma mycoides* subsp. *mycoides* (*Mmm*) is a livestock disease of utmost importance in sub-Saharan Africa. Animals get infected by mycoplasma-containing droplets, leading to acute or subacute disease that may progress into death or chronic malady, although many animals recover. Acute disease is characterized by fever, pleural effusion, severe respiratory distress and a characteristic cough, with a mortality rate of up to 10% in endemic zones [1].

Currently, the control of CBPP in sub-Saharan Africa mainly relies on live vaccines (T1/44 and T1sr) that have their limitations [2]. In the absence of a meaningful small rodent model for *Mmm* infections, research towards improved vaccines relies on studies involving the native host. The current CBPP infection models are based on intratracheal intubation accessed via the nasal or oral cavity, applied blindly via a flexible rubber tube [3] or guided via an bronchoscope [4]. Inoculums reported were pleural effusions from CBPP-positive cattle [5], which are known to contain high concentrations of mycoplasmas [6] or broth cultures containing up to 10^10^ mycoplasmas per mL [7]. Alternatively, in contact transmission has been used to infect cattle, which resembles the natural infection best, but requires donor animals and overall large animal numbers. Recently a novel challenge model for contagious caprine pleuropneumonia was developed, combining aerosol-based intranasal infection and intratracheal infection [8]. This work investigated the use of aerosols containing mycoplasmas to establish an easy to use alternative infection model for CBPP.

**Fig. 1:**
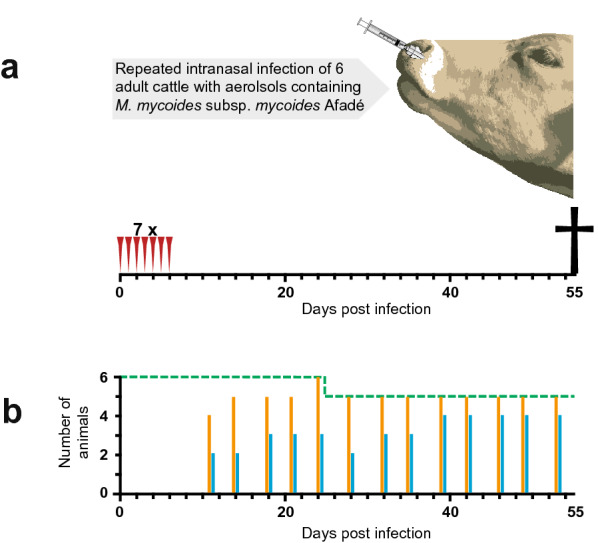
Cartoon displaying the setup of the in vivo trial. **a** Six outbred Boran (*Bos indicus*) heifers were intranasally infected with aerosols of *Mycoplasma mycoides* subsp. *mycoides* strain Afadé on 7 consecutive days and kept up to 55 days post infection. **b** The dashed green line displays the numbers of live animals (only heifer 2 reached endpoint criteria 25 days post infection). The orange and blue bars represent the numbers of animals seroconverted after infection according to the complement fixation test and cELISA, respectively

Six Boran (*Bos indicus*) heifers (No. 1–6) aged 28–37 months at the time of inoculation, weighing 200–260 kg were enrolled in this study. The sample size was determined based on an expectation of 100% morbidity induced by *Mmm* Afadé. We calculated an exact 95% confidence interval around the observed 100% (6/6 animals) morbidity, which indicates a lower limit of 55%, hence we are 95% confident that the true morbidity is at least 55%. The animals were sourced from CBPP-free regions in Kenya. The cattle were vaccinated twice against foot and mouth disease (Fotivax™, Kevevapi, Kenya) at 85 and 66 days before infection and dewormed using Levafas Drench (Norbrook®, UK) at 40 days before infection. All animals tested seronegative for CBPP antibodies using cELISA (IDEXX, Montpellier, France) and complement fixation test (CFT) (CIRAD, Montpellier, France). The layout of the challenge is displayed in Fig. 1a. Two weeks prior to the experimental infection, the cattle were transferred to an animal biosafety level two facility (ABSL-2), where they were kept in one room (25 m^2^) with the ability to move freely and having access to food and water ad libitum. The strain Afadé [9] was cultured as described previously [10], aliquoted and stored at − 80 °C until further use. For the experimental aerosol infection, thawed *Mmm* cultures (5 mL, 5 × 10^8^ colour changing units/mL) were administered equally to each nostril using an atomizer (MAD Nasal™ Intranasal Mucosal Atomization Device, Teleflex®, USA) connected to a lockable glass syringe on seven consecutive days (0–6 dpi). The droplet size generated by the atomizer ranges from 30 to 100 µm. The aerosol was applied during inhaling, took less than 5 min per animal and did not require sedation. The health status of the cattle was monitored daily in the facility. Rectal temperatures exceeding 39 °C were considered as fever. End point criteria were: signs of moderate to severe pain, > 3 consecutive days of high fever (> 40 °C), lateral recumbency for > 1 day, no feeding for > 4 days, breathing frequency of > 50 breaths per minute for > 3 days. The animals were bled twice weekly by jugular vein puncture. Whole blood was used immediately for haematological analysis while serum samples were stored at − 20 °C until further use. Blood parameters were measured using a MEK-6450 Celltac Alpha (Nihon Kohden, Japan) according to vendors’ protocol. Euthanasia was performed as previously described [3] and all necropsies were performed according to standard procedure [11]. Lung and tracheobronchial as well as mediastinal lymph nodes were collected and immediately fixed in neutral buffered 10% formalin (Sigma-Aldrich, USA) at room temperature until further use. The tissues were processed by routine methods and paraffin embedded. Sections of 4 µm were stained with haematoxylin and eosin (HE) for histopathological evaluation. All protocols of this study were designed and performed in strict accordance with the Kenyan legislation for animal experimentation.

Seroconversion was assessed using the CFT [12] and the cELISA [13]. The assays and the results interpretation were performed according to manufacturer’s instructions and the OIE Manual of Diagnostic Tests and Vaccines for Terrestrial Animals [14]. Detailed information on the use of these tests is provided in Additional file 1.

Body temperatures above 39 °C, fever were detected in two out of six animals (2 and 6). In 2 the fever peaked between 23 and 25 dpi with temperatures ranging from 40.3 to 40.5 °C, associated with an overall dullness, coughing, extensive nasal discharge and bleeding from the right nostril (endpoint criteria). This animal was euthanized on 25 dpi. Heifer 6 had mild fever (range 39.1–40.2 °C) for eight consecutive days (27–34 dpi), accompanied by four days of extensive coughing and inappetence (28–31 dpi). The animal did however remain with a slight cough throughout the study (in total 36 days). Extended periods of coughing were also observed in heifers 4 and 5, with coughing being recorded for a total of 35 and 22 days, respectively. For heifer 3, cough was only recorded occasionally. The haematological analysis did not reveal any specific findings (data not shown).

**Fig. 2:**
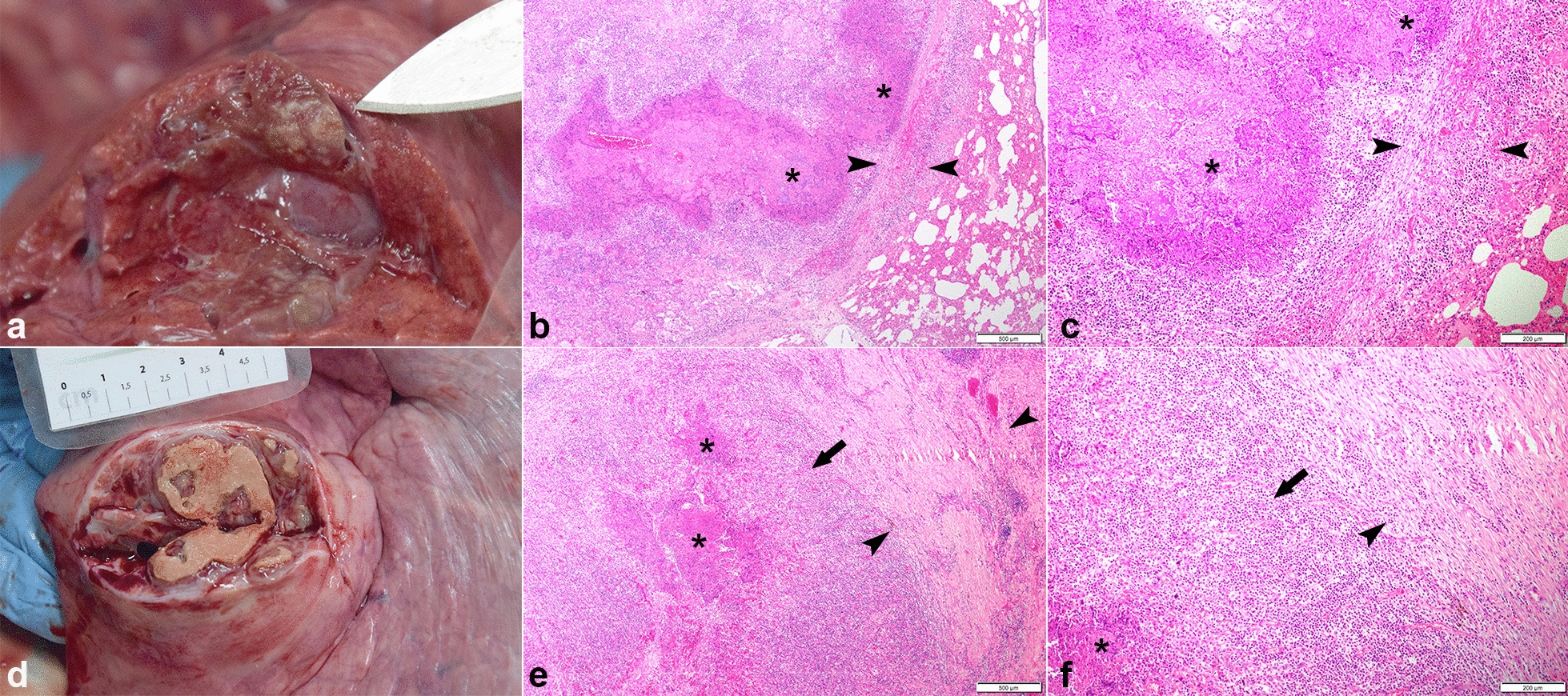
Representative lung lesions and histological findings. **a–c** Heifer 2 showing lesions typical of fibrinous pneumonia (**a**). Histologically **b**, **c** there is central necrosis (*asterisks*) of airways and alveoli extending into interlobular septae (*arrowheads*), which show signs of beginning fibrosis. **d**, **e** Heifer 3 showing more progressed lesions of multifocal fibrinous pneumonia. **d** Histologically there is a thicker zone of inflammatory infiltrate (*arrow*) surrounding the central necrosis (*asterisks*) consisting of neutrophils, lymphocytes and macrophages and marked fibrosis affecting the interlobular septae surrounding the affected lobuli (*arrowheads*)

At necropsy, pleural fluid appeared normal. All animals, except heifer 1, had multifocal fibrinous adhesions between visceral and parietal pleura. There were multiple small consolidated areas in several lung lobes of four animals (Table 1). In heifer 2, these areas appeared as typical lesions of a fibrinous bronchopneumonia with necrosis and haemorrhage of the lung tissue and oedema of the interlobular septae. In animals 3, 4, and 6, several of these lesions were firm. Histopathologically, lung lesions presented as necrotizing and purulent broncho-interstitial pneumonia. Areas of coagulative necrosis and haemorrhage of bronchi, bronchioles and adjacent alveoli were surrounded by many degenerated neutrophils and few lymphocytes, plasma cells and macrophages. In animal 2, there was marked oedema and fibrin exudation. The interlobular septae were inflamed and contained multiple fibrin thrombi in lymphatic vessels. Lungs of heifers 3, 4, and 6 showed more progressive and chronic lesions with areas of necrosis and neutrophil infiltration demarcated by prominent, cell rich connective tissue containing large lymphocytic cell aggregates (Table 1, Fig. 2).

**Table 1:** Summary of clinical and pathological findings

Heifer number	Clinical signs	Macroscopic findings	Histopathological findings
1 (†54 dpi)	None	None	None
2 (†25 dpi)	Cough, fever	Fibrous adhesion: At right diaphragmatic lobe	Necrotizing and suppurative broncho-interstitial pneumonia, entire lobuli affected, extension of necrosis to adjacent lobuli, oedema, fibrin exudation and suppurative inflammation in interlobular septae, fibrin thrombi in interlobular lymphatics, beginning fibrosis at margins of lesions
		Sequester: Seven (1–2 cm in diameter) in right and left diaphragmatic lobe	
3 (†53 dpi)	None	Fibrous adhesion: Between left middle and caudal lobe Between caudal lobe and parietal pleura	Necrotizing and suppurative broncho-interstitial pneumonia, entire lobuli affected, thick, cell rich fibrous connective tissue capsule formation in interlobular septae with lymphocytic infiltration
		Sequester: Two (0.5 and 5 cm in diameter) in left middle and caudal lobe One (3 cm in diameter) in right diaphragmatic lobe	
4 (†54 dpi)	Cough	Fibrous adhesion: Between left caudal lobe and parietal pleura	Necrotizing and suppurative broncho-interstitial pneumonia, entire lobuli affected
		Sequester: One (3 cm in diameter) in left caudal lobe	Thick, cell rich fibrous connective tissue capsule formation in interlobular septae with lymphocytic infiltration
5 (†55 dpi)	Cough	Fibrous adhesion: At right caudal lobe	None
6 (†55 dpi)	Cough, fever	Fibrous adhesion: Multiple at all lung lobes	Necrotizing and suppurative broncho-interstitial pneumonia, entire lobuli affected
		Sequester: Seven (3–12 cm in diameter) in all lung lobes	Thick, cell rich fibrous connective tissue capsule formation in interlobular septae with lymphocytic infiltration

^†^Day of euthanasia; dpi: days post infection

Seroconversion results obtained with different tests varied, which is expected given the different antibody classes detected. According to the CFT, all heifers seroconverted between 11–24 dpi confirming successful infection of all animals. Maximum titres were observed around 40–50 dpi, afterwards titres decreased. Seroconversion measured by cELISA was observed between 11–39 dpi for 5 out of 6 heifers. All results are summarized in Additional file 1. Figure 1b shows the dynamics of the serological response.

Development of an easy to use and reproducible infection model for CBPP has remained one of the major challenges in CBPP vaccine research for years [2]. The infection model tested here builds on the repeated infection via aerosols and the synchronic inoculation of all animals by a defined mycoplasma dose per animal. In contrast to the excellent aerosol-based *Mycoplasma bovis* infection model reported recently [15], the model presented here does not require an infection chamber and can be carried out in resource-poor settings. The fact that just one animal reached endpoint criteria is not surprising as mortality rates of CBPP in the field range from 10 to 20%. All animals infected were prime animals originally intended for breeding purposes without any previous disease record. The pathomorphological changes observed in the five heifers are in agreement to previous findings [16] and textbook knowledge [17]. One animal seroconverted but did not show any lesions 54 dpi.

Intubation is technically demanding. It requires the animals to be sedated and the procedure can be done on standing animals or in lateral recumbency. In most published studies, intubation is performed once, even if repeated intubation over three days were reported [18]. Seroconversion happens earlier in intubated than in in-contact infection [19]. This study confirmed a rather overall late seroconversion in the infection model used here compared to intubated animals [3, 10].

In-contact infection resembles the natural way of disease transmission. Drawbacks of the in-contact challenge model are: (i) it requires an extra group of diseased animals as source of infection to be mingled with the animals to be tested, (ii) efficient transmission happened at a ratio of one infected animal per two naïve animals (1:2), (iii) the onset of infection is different as in the natural setting, making it difficult to compare disease outcomes such as clinical signs and lesions. Therefore, this model requires many animals to provide meaningful data, which is very costly and not in line with 3R guidelines. The present study revealed that the repeated intranasal infection with an aerosolized culture of *Mmm* Afadé caused CBPP. This easy to use experimental infection model avoids an “overchallenge” and will foster pathogenicity, virulence and vaccine studies in the future.

## Supplementary information

**Additional file 1.** Detailed information on the serological, clinical and haematological data.

## Data Availability

All relevant data are included in the manuscript and Additional file 1.

## Abbreviations

CBPP: Contagious bovine pleuropneumonia
dpi: Days post infection
*Mmm*: *Mycoplasma mycoides* subsp. *mycoides*

## References

[1] Di Teodoro G, Marruchella G, Di Provvido A, D'Angelo AR, Orsini G, Di Giuseppe P (2020). Contagious bovine pleuropneumonia: a comprehensive overview. Vet Pathol.

[2] Jores J, Baldwin C, Blanchard A, Browning GF, Colston A, Gerdts V (2020). Contagious bovine and caprine pleuropneumonia: a research community's recommendations for the development of better vaccines. NPJ Vaccines.

[3] Sacchini F, Naessens J, Awino E, Heller M, Hlinak A, Haider W (2011). A minor role of CD4+ T lymphocytes in the control of a primary infection of cattle with *Mycoplasma mycoides* subsp. *mycoides*. Vet Res.

[4] Nkando IG, Wesonga HO, Kuria JK, McKeever D (2010). Assessing the effectiveness of intubation as a challenge model in contagious bovine pleuropneumonia vaccine experiments. Trop Anim Health Prod.

[5] Scacchia M, Sacchini F, Filipponi G, Luciani M, Lelli R, Tjipura-Zaire G (2007). Clinical, humoral and IFNgamma responses of cattle to infection with *Mycoplasma mycoides* var. *mycoides* small colony and attempts to condition the pathogenesis of the infection. Onderstepoort J Vet Res..

[6] Weldearegay YB, Pich A, Schieck E, Liljander A, Gicheru N, Wesonga H (2016). Proteomic characterization of pleural effusion, a specific host niche of *Mycoplasma mycoides* subsp. *mycoides* from cattle with contagious bovine pleuropneumonia (CBPP). J Proteom.

[7] Nkando I, Perez-Casal J, Mwirigi M, Prysliak T, Townsend H, Berberov E (2016). Recombinant *Mycoplasma mycoides* proteins elicit protective immune responses against contagious bovine pleuropneumonia. Vet Immunol Immunopathol.

[8] Liljander A, Sacchini F, Stoffel MH, Schieck E, Stokar-Regenscheit N, Labroussaa F (2019). Reproduction of contagious caprine pleuropneumonia reveals the ability of convalescent sera to reduce hydrogen peroxide production in vitro. Vet Res.

[9] Fischer A, Santana-Cruz I, Hegerman J, Gourle H, Schieck E, Lambert M (2015). High quality draft genomes of the *Mycoplasma mycoides* subsp. *mycoides* challenge strains Afadé and B237. Stand Genomic Sci.

[10] Schieck E, Liljander A, Hamsten C, Gicheru N, Scacchia M, Sacchini F (2014). High antibody titres against predicted *Mycoplasma* surface proteins do not prevent sequestration in infected lung tissue in the course of experimental contagious bovine pleuropneumonia. Vet Microbiol.

[11] Strafuss AC (1988). Necropsy: procedures and basic diagnostic methods for practicing veterinarians.

[12] Etheridge JR, Buttery SH (1976). Improving the specificity and yield of the contagious bovine pleuropneumonia complement fixation test antigen. Res Vet Sci.

[13] Le Goff C, Thiaucourt F (1998). A competitive ELISA for the specific diagnosis of contagious bovine pleuropneumonia (CBPP). Vet Microbiol.

[14] OIE. Contagious bovine pleuropneumonia. In: Manual of diagnostic tests and vaccines for terrestrial animals. Paris: OIE; 2018. p. 1097–112.

[15] Kanci A, Wawegama NK, Marenda MS, Mansell PD, Browning GF, Markham PF (2017). Reproduction of respiratory mycoplasmosis in calves by exposure to an aerosolised culture of *Mycoplasma bovis*. Vet Microbiol.

[16] Sterner-Kock A, Haider W, Sacchini F, Liljander A, Meens J, Poole J (2016). Morphological characterization and immunohistochemical detection of the proinflammatory cytokines IL-1beta, IL-17A, and TNF-alpha in lung lesions associated with contagious bovine pleuropneumonia. Trop Anim Health Prod.

[17] Caswell JL, Williams KJ, Maxie MG (2007). Respiratory System. Jubb, Kennedy, and Palmer’s Pathology of domestic animals.

[18] Miserez R, Pilloud T, Krampe M, Griot C, Bruckner L, Blum J, Poveda JB, Sarris K (1996). Experimental infections in cattle with a European strain and an African strain of *Mycoplasma mvcoides* subsp. *mycoides* SC. Mycoplasmas of ruminants: pathogenicity, diagnostics, epidemiology and molecular genetics.

[19] Dedieu L, Balcer-Rodrigues V, Yaya A, Hamadou B, Cisse O, Diallo M (2005). Gamma interferon-producing CD4 T-cells correlate with resistance to *Mycoplasma mycoides* subsp. *mycoides* S.C. infection in cattle. Vet Immunol Immunopathol..

